# How COVID-19 pandemic period influences on the selected mental health parameters of Polish respondents?

**DOI:** 10.3389/fpsyg.2023.1126471

**Published:** 2023-05-25

**Authors:** Szymon Florek, Magdalena Piegza, Paweł Dębski, Piotr Gorczyca, Robert Pudlo

**Affiliations:** Department of Psychiatry, Faculty of Medical Sciences in Zabrze, Medical University of Silesia in Katowice, Katowice, Poland

**Keywords:** COVID-19, mental health, anxiety, aggression, alcohol consumption, adaptation

## Abstract

**Introduction:**

There are many different articles about COVID-19 pandemic period and its influence on people and their behavior. Nevertheless, there is little research on the slightly later period of the pandemic, that is, the time when specific adaptation mechanisms in society should start to take place.

**Methods:**

Our research was conducted by means of an online survey. Four hundred and eighty five adults participated, including 349 (71.96%) women and 136 (28.04%) men. The Buss-Perry aggression scale, Alcohol Use Disorders Identification Test and Generalized Anxiety Disorder 7 scale were used. The results were statistically processed using Statistica 13.3 software.

**Results:**

Within the study population, positive correlations were noted between anxiety and generalized aggression, anger, hostility, physical and psychological aggression. In the female group, anxiety correlates positively with generalized aggression, anger, hostility, verbal and physical aggression. Among male subjects, anxiety correlates positively with aggression, anger, and hostility. Alcohol consumption has a significant association with verbal aggression. Statistically, more women experience anxiety, more men have inflated scores on the AUDIT scale and on verbal and physical aggression. Younger people are more likely than older people to experience anxiety and have inflated scores on hostility. Those with secondary education scored significantly higher on the GAD-7 scale and the aggression scale (and all subscales except anger) compared to respondents with higher education.

**Discussion:**

As a result of adaptation to the COVID-19 pandemic, anxiety is no longer a factor in increased evels of alcohol consumption. The pandemic has not affected differences in alcohol consumption between men and women. The presence of a positive correlation between anxiety and aggression and the sociodemographic structure of those characterized by increased aggression are also unchanged. Anxiety directly influences aggressive behavior in a relatively strong way. Appropriate health-promoting measures should be implemented to protect the public from the negative effects of the COVID-19 pandemic.

## 1. Introduction

COVID-19 is an infectious disease whose first cases were described in Wuhan, China (Wu et al., 2020; Zhou et al., 2020). Its most common symptoms include fever, fatigue, dry cough, muscle pain, and dyspnea. Gastrointestinal symptoms are also important and may precede the others (Wang et al., 2020). In addition to these, the SARS-CoV-2 virus can also attack many other organs and systems such as the heart, kidneys, liver, and nervous system—both central and peripheral (Renu et al., 2020; Andalib et al., 2021). Symptoms originating from the latter include headache, hyposmia or anosmia, taste disturbances, encephalopathy, epilepsy, impaired consciousness, cerebrovascular events (both haemorrhagic and ischemic stroke), meningitis and encephalitis, or even Guillain-Barre syndrome. Long-term complications are also highly likely, but have not yet been fully studied due to the relatively short duration of the pandemic (Abboud et al., 2020). It cannot be taken out of the equation that they will also include mental health. During the COVID-19 pandemic, many studies have also been conducted to determine its impact on many different aspects of life in different socio-demographic groups, also among Polish students (Almeida et al., 2020; Chen et al., 2021; Jones et al., 2021; Juchnowicz et al., 2021; Sitarz et al., 2021; Thatrimontrichai et al., 2021). Research among the latter group indicates elevated levels of anxiety, stress and depressive symptoms during the greatest constraints of the lockdown period. However, as the second cross-sectional study showed, the students coped in different ways with the symptoms they encountered, but did not avoid them (Juchnowicz et al., 2021; Sitarz et al., 2021). Despite the pandemic adaptation mechanisms observed in society, it is difficult to find scientific studies on this topic. For this reason, the authors of this article decided to conduct a relevant project.

Adaptation, related disorders or adaptability can be considered on many different paths. In the context of illness, two of the most important can be distinguished. The first relates to the individual’s response to the illness, and the second, more global, relates to the general pattern of response to severe stress. According to accepted theory, everyone has a certain level of tension in which he or she feels comfortable—both emotionally and in terms of certain behaviors, standards of living, etc. Any stimulus that disrupts this, triggers the body to adapt to the new situation and—if possible—to take it as a new “level zero.” In the case of illness or severe stress, this mechanism can be disrupted producing a general adaptation syndrome. It is always characterized by the same three phases of the stress response, these being alarm response, resistance and exhaustion. In a way, the reaction to serious illness created for health psychology fits into this theory, in which one can distinguish the phases: a search for meaning, a search for mastery and a process of self-enhancement (Odgen, 2004; Myers, 2010). This is reflected in psychiatry, where one can find diagnoses in the field of adaptive reaction within some symptoms like anxiety, restlessness, depression, tension, tearfulness or sleep disorders (Gałecki and Szulc, 2018). In the case of prolonged stress, there is no clear time limit set for when full adaptation to the surrounding conditions would occur—this is due to the fact that every organism reacts—within the framework presented—differently.

Research on the co-occurrence of anxiety and alcohol dependence was noted as early as the ‘80s (Wilson, 1998), and it is contemporaneous with a variety of careful analyses, including biochemical analyses, which have revealed a likely common source of anxiety and alcohol dependence in amygdala dysfunction. This may explain the abuse of alcohol while experiencing high anxiety, as well as the occurrence of anxiety as a symptom of abstinence syndrome in alcohol dependence (Gilpin et al., 2015). In view of the facts presented, it seems that alcohol consumption, on the one hand, may be modified against the background of adaptive response to the COVID-19 pandemic, and on the other hand—according to numerous reports, it may have already been modified by the very presence of the pandemic and the restrictions associated with lockdown (Ramalho, 2020). Moreover, numerous studies conducted so far have shown that increased alcohol consumption increases aggression in various mechanisms. Interestingly, as Kuypers reports, this mechanism is not so obvious and so well researched for other psychoactive substances (Kuypers et al., 2020). It is very likely that the COVID-19 pandemic also contributed to the increase in violence, as the study found that such a mechanism occurs as a result of natural disasters (Molyneaux et al., 2019). What is more—the cited study concerns the effects of disasters, so the increased level of aggression in this mechanism seems to be delayed in relation to the COVID-19 pandemic.

A separate, but equally important, issue is the inclusion of the parameters under study in a comparative analysis designating specific socio-demographic groups. In addition to disrupting the interrelationships between the variables studied, the pandemic may also have left its mark on the reduction, or increase, of differences between men and women, younger and older people. Similar studies, but at a slightly different time, were conducted, for example, in Estonia (Tamson et al., 2022), and the variability of mental health status during the COVID-19 pandemic among different demographic groups (Blanchflower and Bryson, 2022).

The aim of our study was to identify the links between anxiety, aggression and the level of alcohol consumption over a period of more than 2 years since the first case of COVID-19 was diagnosed in Poland. This period was selected due to the fact that, according to Heitzman, the specific form of acute stress response associated with COVID-19 should last much longer than that adopted in accordance with the ICD-10 criteria (World Health Organisation, 1993; Heitzman, 2020). Therefore, it is difficult to determine the exact time of society’s adaptation to the new situation, and research related to this phenomenon is increasingly needed. We conducted our study from 5 February 2022 to 6 March 2022. During this time, 650,709 new cases of COVID-19 were recorded in Poland, 6,229 people died, and 957,913 people recovered. These data come from the statistics kept by the Polish Ministry of Health.

## 2. Materials and methods

Our project was entirely carried out *via* the Internet in order to obtain as many respondents as possible from various regions of Poland. The survey form was shared *via* social media sites such as Facebook. A total of 1,267 respondents completed the online survey, but only 485 met the inclusion criteria, of whom 349 (71.96%) were women and 136 (28.04%) were men. The exact socio-demographic structure of the studied population is presented in Table 1. At this point, it should be noted that the presented study is a separate project from previously conducted similar research (Florek et al., 2021). Before sending the questionnaires, the authors contacted the Bioethics Committee at the Medical University of Silesia in Katowice in order to obtain its opinion whether this project requires the appropriate consent. Having examined our letter, the committee decided that due to the nature of the examination, such consent was not required.

**Table 1:** The socio-demographic structure of the studied population.

	Number of respondents	Percentage value (%)
Total	485	100
**Sex**		
Female	349	71.96
Male	136	28.04
**Domicile**		
A city with over 200,000 inhabitants	224	46.19
City with 50,000–200,000 inhabitants	113	23.30
A town with less than 50,000 inhabitants	70	14.43
Village	78	16.08
**Age**		
55+	20	4.12
36–55	142	29.28
26–35	177	36.49
18–25	146	30.10
**Education**		
Higher	328	67.63
Secondary	141	29.07
Vocational	2	0.41
Primary	10	2.06
No answer	4	0.82

The criteria for inclusion in the study were informed consent to participate in the project and the age of 18. The respondents gave their consent by accepting a detailed instruction placed at the beginning of the survey. It contained all the necessary information, including a notification of the possibility of resigning from participation in the project at any stage without any consequences. No personal data was collected for the purposes of the study, and multiple participation in the study was eliminated through a control question that had to be answered appropriately. The criteria for exclusion from the study included therapy with a psychiatrist in the last 6 months prior to the survey, as well as the presence of events within 12 months that caused major changes in the respondents’ lives. Relevant questions regarding the presented variables were placed in the initial survey, and all people who met them were excluded.

In this study, psychometric scales were used exactly as in the project carried out in 2020 (Florek et al., 2021). The GAD-7 scale used to measure the intensity of anxiety contains 7 questions, on which respondents answer on a 4-point scale, and the result is their simple sum (Spitzer et al., 2006). The AUDIT is the screening test to initially identify alcohol dependence and consists of 10 questions. Answers are on a three- or five-point scale, the simple sum of which is the final score (Saunders et al., 1993). To test the level of aggression, the Polish adaptation of the Buss-Perry Aggression Scale was used, in which 29 statements were included, and the respondents expressed their attitude toward them on a five-point scale. The results are obtained by inverting the scores of two questions and the sum of the remaining ones for the full scale of generalized aggression and by summing up the points from selected questions for specific subscales (Buss and Perry, 1992; Siekierka, 2005). Statistical analysis was performed with the use of Excel 365 and Statistica 13.3. The owner of the software license is the Medical University of Silesia in Katowice. In order to assess the normal distribution for the examined variables, a graphical plot was made each time and the Shapiro–Wilk test was applied. Due to the presence of abnormal distributions, the Spearman’s rank correlation test was used to calculate the correlation. For generalized aggression, a linear regression model was used. An analysis of variance, the assumptions of regression linearity were checked using the residual distribution analysis, the Durbin-Watson test, and the variance stability was confirmed on the appropriate graph. Comparative analyses for normal distributions were carried out using the Student’s t-test, while for variables with non-normal distributions, the Mann Whitney *U*-test was used. For comparisons of more than 2 groups of independent variables, a Kruskal-Wallis ANOVA was used. The chi-square test was used for variables obtained after converting the raw scale scores using the respective norms. Statistical considerations were made at the significance level of α < 0.05.

## 3. Results

Within the entire study population, there was a positive correlation between anxiety and generalized aggression, anger and hostility, as well as weak positive correlation between anxiety and physical and verbal aggression. The discussed relationships are presented in Table 2. Among the studied linear regressions, one statistically significant regression model of generalized aggression in the light of anxiety was obtained, which is presented in Table 3.

**Table 2:** Relationships between the severity of anxiety, the level of alcohol consumption and aggression.

*N* = 485	Anxiety	Alcohol	Generalized aggression	Verbal aggression	Physical aggression	Anger	Hostility
Anxiety	1.000	−0.067	**0.369** ^*^	**0.110** ^*^	**0.103** ^*^	**0.348** ^*^	**0.467** ^*^
Alcohol		1.000	0.020	0.004	−0.018	0.032	0.015
Generalized aggression			1.000	**0.674** ^*^	**0.704** ^*^	**0.841** ^*^	**0.789** ^*^
Verbal aggression				1.000	**0.442** ^*^	**0.524** ^*^	**0.330** ^*^
Physical aggression					1.000	**0.469** ^*^	**0.379** ^*^
Anger						1.000	**0.546** ^*^
Hostility							1.000

* Statistically significant result at *p* < 0.05.

**Table 3:** Regression model of generalized aggression toward severity of anxiety.

Predictor	b	b SE	Beta	Beta SE	t	*p*
Constant	60.080	1.325	---	---	45.356	**<0.001** ^*^
Anxiety	0.142	1.219	0.042	0.364	8.582	**<0.001** ^*^

* Statistically significant result at *p* < 0.05; SE, Standard Error; Corr. R-squared = 0.1323; *F*(1.483) = 73.643; *p* < 0.001; error of estimation = 16.373.

In the group of women, anxiety correlated positively with generalized aggression, anger and hostility, and weakly positively correlated with verbal and physical aggression, which basically corresponds to the entire study population. Among male respondents, only a moderately positive correlation with aggression and anger, and a strongly positive correlation with hostility was noted in terms of anxiety. An average negative correlation between alcohol consumption and verbal aggression was also shown. These results are presented in Table 4. Female respondents showed higher levels of anxiety than male respondents. In contrast, alcohol consumption and levels of physical and verbal aggression were higher in the male study group, as shown in Table 5. The data between the other parameters—generalized aggression, hostility and anger—were also analyzed, but no statistically significant differences became apparent.

**Table 4:** Relationships between the severity of anxiety, the level of alcohol consumption and aggression among men.

*N* = 136	Anxiety	Alcohol	Generalized aggression	Verbal aggression	Physical aggression	Anger	Hostility
Anxiety	1.000	−0.096	**0.384** ^*^	0.000	0.150	**0.330** ^*^	**0.557** ^*^
Alcohol		1.000	−0.151	**−0.213** ^*^	−0.049	−0.077	−0.128
Generalized aggression			1.000	**0.566** ^*^	**0.694** ^*^	**0.848** ^*^	**0.759** ^*^
Verbal aggression				1.000	**0.355** ^*^	**0.460** ^*^	**0.169** ^*^
Physical aggression					1.000	**0.481** ^*^	**0.342** ^*^
Anger						1.000	**0.522** ^*^
Hostility							1.000

* Statistically significant result at *p* < 0.05.

**Table 5:** Comparative analysis of the standardized scores of the scales used for the groups of men and women, *p* < 0.05.

*N* = 485	Men (*n* = 136)	Women (*n* = 349)	*p*
**Anxiety**						**<0.001** ^*^
No anxiety (0–4 points)	60	87	
Mild anxiety (5–9 points)	45	145	
Moderate anxiety (10–14 points)	19	71	
Serious anxiety (15–21 points)	12	46	
**Alcohol consumption**						**<0.01** ^*^
Low risk of dependence (0–7 points)	101	311	
Risky consuming (8–15 points)	29	33	
Harmful consuming (16–19 points)	3	2	
Alcohol addiction (20–40 points)	3	3	
**Verbal aggression**						**<0.05** ^*^
Very low scores	44	148	
Low scores	0	0	
Reduced scores	28	58	
Average scores	15	24	
Elevated scores	6	31	
High scores	7	24	
very high scores	36	64	
**Physical aggression**						**<0.01** ^*^
Very low scores	69	241	
Low scores	23	35	
Reduced scores	7	14	
Average scores	14	21	
Elevated scores	5	7	
High scores	9	8	
Very high scores	9	23	

* Statistically significant result at *p* < 0.05; normalization of the aggression scale is based on 10 values not included in the table.

**Figure 1:**
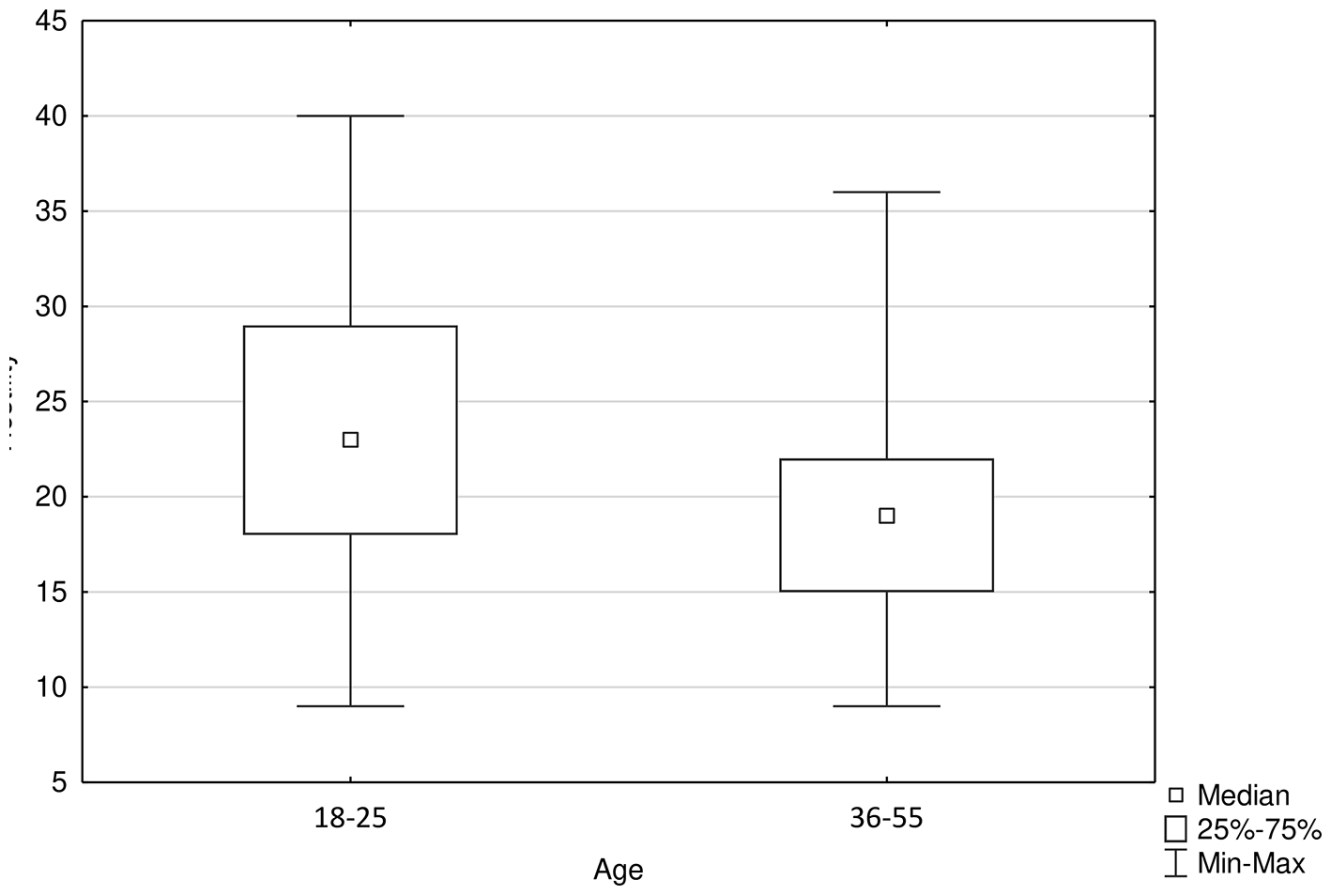
Significantly higher levels of hostility among 18–25 years old compared to 36–55 old respondents, *p* < 0.001.

When analyzing the group of people aged 18–25, anxiety correlations were noticed, corresponding to those shown among the surveyed men. There was no correlation between alcohol consumption and other scales. The correlations among respondents aged 26–35 generally corresponded to those reported for the entire study population. Interesting results were obtained among people aged between 36 and 54, where generally correlations are similar to the men group excluding alcohol. Due to the small size of the group of people over 55 (19 respondents), the correlation analysis was abandoned due to the high risk of obtaining results that could be misleading. In the comparative analysis, the levels of anxiety and generalized aggression were significantly higher among younger respondents (18–25 years) than among the other age groups (Table 6). Moreover, in the analysis of raw hostility scores, hostility was higher among respondents of this age relative to respondents aged 36–55 years (Figure 1).

**Table 6:** Comparative analysis of standardized GAD-7 scale scores and generalized aggression when the study population was divided by age, *p* < 0.05.

*N* = 465	18–25 years	26–35 years	36–55 years	*p*
	(*n* = 146)	(*n* = 177)	(*n* = 142)	
**Anxiety**				**<0.05** ^*^
No anxiety (0–4 points)	38	50	54	
Mild anxiety (5–9 points)	49	72	59	
Moderate anxiety (10–14 points)	33	35	19	
Serious anxiety (15–21 points)	26	20	10	
**Generalized aggression**				**<0.05** ^*^
Very low scores	70	87	83	
Low scores	13	23	24	
Reduced scores	6	9	5	
Average scores	17	17	13	
Elevated scores	2	6	5	
High scores	9	9	1	
Very high scores	29	26	11	

^*^Statistically significant result at *p* < 0.05; normalization of the aggression scale is based on 10 values not included in the table.

A fairly strong positive correlation between anxiety and aggression and anger, and a strong correlation with hostility was identified among rural residents (Table 7). It is similar among city dwellers between 50,000 and 200,000 inhabitants (Table 8). On the other hand, among respondents from cities with less than 50,000 inhabitants, anxiety has a weak positive correlation with anger and hostility (Table 9). The correlations among inhabitants of cities with more than 200,000 inhabitants look slightly different—among them, anxiety correlates positively with aggression, anger and quite strongly with hostility, and alcohol consumption has a weak negative correlation with physical aggression, which is presented in Table 10. Comparative analysis did not reveal any significant differences between the study groups.

**Table 7:** Relationships between the severity of anxiety, the level of alcohol consumption and aggression among rural residents.

*N* = 136	Anxiety	Alcohol	Generalized aggression	Verbal aggression	Physical aggression	Anger	Hostility
Anxiety	1.000	0.024	**0.474** ^*^	0.203	0.175	**0.411** ^*^	**0.568** ^*^
Alcohol		1.000	0.150	0.120	0.109	0.163	0.035
Generalized aggression			1.000	**0.710** ^*^	**0.735** ^*^	**0.898** ^*^	**0.801** ^*^
Verbal aggression				1.000	**0.594** ^*^	**0.556** ^*^	**0.366** ^*^
Physical aggression					1.000	**0.584** ^*^	**0.361** ^*^
Anger						1.000	**0.670** ^*^
Hostility							1.000

* Statistically significant result at *p* < 0.05.

**Table 8:** Relationships between the severity of anxiety, the level of alcohol consumption and aggression among city dwellers with between 50,000 and 200,000 inhabitants.

*N* = 136	Anxiety	Alcohol	Generalized aggression	Verbal aggression	Physical aggression	Anger	Hostility
Anxiety	1.000	−0.037	**0.363** ^*^	0.044	0.036	**0.372** ^*^	**0.483** ^*^
Alcohol		1.000	0.072	−0.013	−0.009	0.151	0.013
Generalized aggression			1.000	**0.648** ^*^	**0.610** ^*^	**0.752** ^*^	**0.736** ^*^
Verbal aggression				1.000	**0.266** ^*^	**0.517** ^*^	**0.268** ^*^
Physical aggression					1.000	**0.294** ^*^	**0.312** ^*^
Anger						1.000	**0.338** ^*^
Hostility							1.000

* Statistically significant result at *p* < 0.05.

**Table 9:** Relationships between the severity of anxiety, the level of alcohol consumption and aggression among city dwellers with less than 50,000 inhabitants.

*N* = 136	Anxiety	Alcohol	Generalized aggression	Verbal aggression	Physical aggression	Anger	Hostility
Anxiety	1.000	−0.147	0.201	0.160	−0.001	**0.281** ^*^	**0.246** ^*^
Alcohol		1.000	0.151	0.165	0.191	0.020	0.119
Generalized aggression			1.000	**0.744** ^*^	**0.742** ^*^	**0.839** ^*^	**0.843** ^*^
Verbal aggression				1.000	**0.532** ^*^	**0.616** ^*^	**0.482** ^*^
Physical aggression					1.000	**0.455** ^*^	**0.512** ^*^
Anger						1.000	**0.603** ^*^
Hostility							1.000

* Statistically significant result at *p* < 0.05.

**Table 10:** Relationships between the severity of anxiety, the level of alcohol consumption and aggression among city dwellers with more than 200,000 inhabitants.

*N* = 136	Anxiety	Alcohol	Generalized aggression	Verbal aggression	Physical aggression	Anger	Hostility
Anxiety	1.000	−0.099	**0.381** ^*^	0.099	0.131	**0.331** ^*^	**0.470** ^*^
Alcohol		1.000	−0.064	−0.059	**−0.150** ^*^	−0.040	0.000
Generalized aggression			1.000	**0.657** ^*^	**0.726** ^*^	**0.846** ^*^	**0.789** ^*^
Verbal aggression				1.000	**0.453** ^*^	**0.485** ^*^	**0.304** ^*^
Physical aggression					1.000	**0.501** ^*^	**0.394** ^*^
Anger						1.000	**0.568** ^*^
Hostility							1.000

* Statistically significant result at *p* < 0.05.

Due to the size of individual groups, it was decided to analyze only people with secondary and higher education (see Table 1). Among people with secondary education, anxiety correlates positively with aggression, anger and hostility, while in the group of people with higher education, there is a moderate correlation of anxiety with aggression and anger. What is more, anxiety correlates weakly positively with physical aggression and quite positively with hostility in this group. In the light of the previously presented results, these results can be assumed to be the same as for the entire study group. The situation is similar with the division into health care workers, among whom anxiety correlates positively with aggression, anger, and strongly positively with hostility. The correlations of anxiety in the group of non-health care workers were moderate and positive with aggression, anger, and moderate and strongly with hostility. Taking these two divisions of the study population into account, the comparative analysis did not reveal any significant differences between health care workers and people of different occupation. However, analysis of the raw data from the scales highlighted statistically significant differences between those with secondary and tertiary education, as shown in Table 11.

**Table 11:** Comparative analysis of the raw scores of the anxiety and aggression scales when the study population is divided by education.

Variable	Secondary education *n* = 141			Higher education *n* = 328			Z	Cohen’s d	*p*
	Mean	SD	Median	Mean	SD	Median			
Anxiety	8.752	5.531	7.000	7.134	5.024	6.000	−2.929	−0.312	**<0.01** ^*^
Generalized aggression	72.461	17.885	72.000	67.595	16.903	67.000	−2.650	−0.283	**<0.01** ^*^
Verbal aggression	14.709	3.947	14.000	13.680	3.785	13.000	−2.473	−0.268	**<0.05** ^*^
Physical aggression	17.227	5.527	16.000	16.018	5.226	15.000	−2.364	−0.227	**<0.05** ^*^
Anger	17.596	6.175	18.000	17.460	6.012	17.000	−0.224	−0.022	0.823
Hostility	22.929	7.186	23.000	20.436	6.746	20.000	−3.338	−0.362	**<0.001** ^*^

^*^Statistically significant result at *p* < 0.05.

## 4. Discussion

When analyzing the relationships between the parameters studied, it can be seen that the level of alcohol consumption is practically irrelevant in the study population. However, with reference to the 2020 study, a strengthening of the correlation between anxiety and aggression and its components becomes apparent (Florek et al., 2021). A decrease in the role of alcohol consumption is also evident in the regression analysis, where only the effect of anxiety level on generalized aggression was shown. Interestingly, numerous studies conducted to date indicate an increase in alcohol consumption levels during the COVID-19 pandemic (Murthy and Narasimha, 2021; Roberts et al., 2021). As reported by Grossman et al. the increased alcohol consumption during this period in the United States was primarily influenced by stress (Grossman et al., 2020). It is worth noting, however, that all the studies mentioned refer to the period of development of the pandemic—i.e., mainly 2020. There is little data in the literature on the later period. One study of US adolescents found that by June 2021, the drinking patterns created with the onset of the pandemic had already virtually disappeared (Pelham 3rd et al., 2022). Moreover, as a large meta-analysis has shown, there was a decline in global alcohol consumption levels during the pandemic, and evidence of an increase exists among those already abusing alcohol (Kilian et al., 2022). In light of the research presented here, our results appear to fit into global mechanisms of change in alcohol consumption. Most studies to date indicate that alcohol increases levels of aggression, particularly in men (Giancola et al., 2002, 2009). Our study shows the opposite, as alcohol consumption among male respondents correlates negatively with the intensity of verbal aggression, and negatively with physical aggression among residents of the largest cities. Referring to many years of research on aggression and its mechanisms, it should be noted that the indirect effect of alcohol has been best proven. In fact, the general aggression model developed is based on this effect. At this point, it should be noted that indirect effects are distinguished by the fact that alcohol makes it easier, so to speak, to provoke a person into aggressive behavior, while ethanol itself even reduces the level of both physical and psychological arousal that can stimulate aggressive behavior (Bushman, 1997; Anderson and Bushman, 2002). The latter property may cause the negative correlations highlighted in the different groups. In addition, it is possible that the continuing tension associated with the COVID-19 pandemic and any restrictions has lowered the threshold for provocation to aggression by different from alcohol mechanisms. However, this phenomenon would be quite new and requires further research. It should be emphasized, however, that the mechanisms discussed in the context of the presented results of our study only serve to illustrate the potential causes of certain correlations. Indeed, no statistically significant regression of alcohol consumption was evident, in contrast to the previous study (Florek et al., 2021). In the comparative analysis of alcohol consumption levels, it is noteworthy that more male respondents consume larger amounts of alcohol statistically. This fact is not surprising, as it fits the characteristics of the alcohol consumption pattern (Manwell et al., 2002) that is present in Poland and coincides with the reports of a study conducted at the beginning of the COVID-19 pandemic (Florek et al., 2022).

Undoubtedly, the correlation between aggression and anxiety is very important. As mentioned in the introduction, many researchers have wondered about the mutual influence of these two parameters. In our study, we showed within the whole study population a positive correlation between anxiety and generalized aggression and all its subscales. This correlation should not come as a surprise, as Chung reports that it already occurs in adolescents (Chung et al., 2019). However, interesting results were obtained among male respondents, those aged 36–55 years and residents of cities with more than 200,000 inhabitants, where anxiety does not correlate with verbal and physical aggression. These findings correlate with a study of drug treatment center inpatients, who were sociodemographically characterized by racial diversity, most of whom were single, unemployed, with low incomes and no more than a high school education (Dixon et al., 2017). In addition, other studies suggest low levels of empathy and fear of being judged as reasons for expressing aggression precisely in the form of anger and hostility rather than physical or verbal aggression (Loudin et al., 2003; Hanby et al., 2012). In light of the research presented here, however, it is reasonable to assume that the relationship between anxiety and aggression—however complex—did not change during the COVID-19 pandemic. In the comparative analysis, physical and verbal aggression are significantly higher in the male group. This fact, however, is not surprising anyway and, as a study among minors shows, it is already present in adolescents as young as 15 years old (Österman et al., 1998). It is noteworthy that in our survey, hostility in terms of raw scores came out significantly higher in younger people aged 18–25 compared to those aged 36–55. Furthermore, those with a secondary education had significantly higher levels of generalized aggression, physical aggression, verbal aggression and hostility and non-significantly anger compared to those with a higher education. With reference to the project carried out at the beginning of the COVID-19 pandemic in Poland, it can be concluded that these differences remained essentially unchanged (Florek et al., 2022). In terms of physical and verbal aggression they have strengthened, while in terms of anger they have weakened. However, this does not seem to be part of the adaptability to the COVID-19 pandemic discussed in this article.

The last parameter analyzed is anxiety intensity. It was mostly discussed above in the context of its influence on the level of alcohol consumption or aggression. However, it is worth paying attention to the regression model, which clearly shows the influence of anxiety on the level of generalized aggression and is essentially unchanged from the previous study (Florek et al., 2021). In the context of the research conducted to date (Mcmurran, 2011; Parrott et al., 2012), it seems surprising that this is the only statistically significant regression, as it is natural for both alcohol consumption and aggression to increase under the influence of anxiety. Indeed, there is evidence that anxiety and aggression may be regulated by the same neurohormones. Although the results of a study conducted in this regard were not conclusive in all circumstances, researchers have confirmed this relationship (Neumann et al., 2010). Furthermore, Granic’s work highlights the frequent co-occurrence of anxiety and aggression in children (Granic, 2014). Given these reports, one may wonder whether the COVID-19 pandemic has not reinforced the most primary link among people, which is anxiety and aggression, bypassing an indirect factor like alcohol consumption. The higher severity of anxiety among females is not surprising, as there are numerous publications reporting on this variable (Bahrami and Yousefi, 2011; McLean et al., 2011; Bandelow and Michaelis, 2015). Furthermore, a study of 100 Iranian secondary school students shows that higher levels of anxiety are already found in girls aged 15–18 years (Bahrami and Yousefi, 2011). On the other hand. It seems to be an interesting difference in the severity of anxiety when dividing the group by age. It turned out that it is the youngest individuals who are most likely to experience anxiety. Studies to date (outside of the pandemic period) are inconclusive and indicate either that anxiety is highest in middle age (Bandelow and Michaelis, 2015) or that they do not specify age indicating a number of other factors modeling anxiety (Jorm, 2000). Compared to the study conducted during the COVID-19 pandemic, it should be noted that no significant difference in anxiety was highlighted between the study groups at that time. The situation is similar with the division by educational level. During the previous study, there were also no differences highlighted by conducting this one. This may mean that the COVID-19 pandemic has strong impact on anxiety in young people who are studying or who are in the process of studying or looking for their first job, i.e., at extremely important moments in their lives. In the light of the Norwegian study, this hypothesis seems quite plausible, as the researchers concluded that factors associated with higher education can protect against anxiety and depression practically throughout life (Bjelland et al., 2008), meaning that in a stressful situation such as the COVID-19 pandemic, people with lower education will be more susceptible to stress.

### 4.1. Limitations and strengths

Our survey has its limitations. It should be noted that it was conducted *via* the Internet. Although this methodology is increasingly used in various scientific works—including psychological and psychiatric research—it has its drawbacks. These include, first and foremost, the impossibility of observing the research participant and thus answering their questions. On the other hand, thanks to this form of survey, it was possible to reach a really large number of respondents, which, in the opinion of the authors, would not have been possible if the survey had been conducted in the traditional way. It is worth noting that people over 55 years of age were excluded from the analyses with the age division of the surveyed population due to the small size of this group (19 respondents). Of course, this is most likely due to the small percentage of older people using the Internet. However, this also has its advantages, as our study focuses on people of working age, i.e., those who may have been most affected by the pandemic due to, for example, changes in the nature of work, working hours, or certain redundancy movements. There was also a certain disproportionality, which could distort the analyses, in the division by place of residence. The largest group was made up of people living in cities with more than 200,000 inhabitants (208 respondents), while the smallest number of respondents were rural residents—69 people. Such a situation may have been due to the channels of transmission of information, as it was difficult for the authors to reach Internet forums or other sites that may be related to rural life in the broadest sense. Here, it is also worth noting that the pandemic probably made the least difference in the countryside—there was only a short period of time when people were not allowed to go out even into the forests in Poland. Outside of it—farms were generally able to function without much change. There was a similar problem when dividing the population into health care workers and others—here there were only 126 health care workers (and 359 others). Another observation concerns education. People with less than secondary education were missing from the study population. Perhaps people with lower education were not interested in participating in the scientific study or, as in the case of the rural population, the authors were unable to reach the websites that these people visit. For this reason, analyses of differences and correlations were only carried out in the groups of people with secondary and higher education.

Taking into account the above-mentioned limitations, it should be clearly stated that the survey was not conducted on a representative group of Polish society, and therefore—the results should not be translated to a wider group of society outside the respondents taking part in the survey. In addition, it is worth noting that the questionnaire did not include questions about the survey participants’ contact with the COVID-19 disease. On the other hand, it should be emphasized that the disease and its limitations affected the vast majority of the population, as confirmed by numerous publications cited in this article (Almeida et al., 2020; Chen et al., 2021; Jones et al., 2021; Juchnowicz et al., 2021; Sitarz et al., 2021; Thatrimontrichai et al., 2021).

## 5. Conclusion

1. As a result of adaptation to the COVID-19 pandemic, anxiety is no longer a factor in increased levels of alcohol consumption. Furthermore, increased alcohol consumption is not associated with increased aggressive behavior.
2. The pandemic has not affected differences in alcohol consumption between men and women. Still, more alcoholic beverages are consumed by men. Moreover, the presence of a positive correlation between anxiety and aggression and the sociodemographic structure of those characterized by increased aggression are also unchanged.
3. Anxiety directly influences aggressive behavior in a relatively strong way.

## Data availability statement

The raw data supporting the conclusions of this article will be made available by the authors, without undue reservation.

## Ethics statement

The studies involving human participants were reviewed and approved by Bioethics Committee of the Silesian Medical University in Katowice. The patients/participants provided their written informed consent to participate in this study.

## Author contributions

PD, SF, MP, and RP contributed to conception, design of the study, and wrote sections of the manuscript. SF and MP organized the database and wrote the first draft of the manuscript. SF and PD performed the statistical analysis. All authors contributed to manuscript revision, read, and approved the submitted version. All authors contributed to the article and approved the submitted version.

## Conflict of interest

The authors declare that the research was conducted in the absence of any commercial or financial relationships that could be construed as a potential conflict of interest.

## Publisher’s note

All claims expressed in this article are solely those of the authors and do not necessarily represent those of their affiliated organizations, or those of the publisher, the editors and the reviewers. Any product that may be evaluated in this article, or claim that may be made by its manufacturer, is not guaranteed or endorsed by the publisher.
